# Marker-assisted selection for transfer of QTLs to a promising line for drought tolerance in wheat (*Triticum aestivum* L.)

**DOI:** 10.3389/fpls.2023.1147200

**Published:** 2023-07-21

**Authors:** V. P. Sunilkumar, Hari Krishna, Narayana Bhat Devate, Karthik Kumar Manjunath, Divya Chauhan, Shweta Singh, Nivedita Sinha, Jang Bahadur Singh, Prakasha T. L., Dharam Pal, M. Sivasamy, Neelu Jain, Gyanendra Pratap Singh, Pradeep Kumar Singh

**Affiliations:** ^1^Indian Agricultural Research Institute, ICAR, New Delhi, India; ^2^National Bureau of Plant Genetic Resources, ICAR, New Delhi, India

**Keywords:** HD3086, drought tolerance, foreground selection, QTLs, MABB

## Abstract

Wheat crop is subjected to various biotic and abiotic stresses, which affect crop productivity and yield. Among various abiotic stresses, drought stress is a major problem considering the current global climate change scenario. A high-yielding wheat variety, HD3086, has been released for commercial cultivation under timely sown irrigated conditions for the North Western Plain Zone (NWPZ) and North Eastern Plain Zone NEPZ of India. Presently, HD3086 is one of the highest breeder seed indented wheat varieties and has a stable yield over the years. However, under moisture deficit conditions, its potential yield cannot be achieved. The present study was undertaken to transfer drought-tolerant QTLs in the background of the variety HD3086 using marker-assisted backcross breeding. QTLs governing Biomass (BIO), Canopy Temperature (CT), Thousand Kernel Weight (TKW), Normalized Difference Vegetation Index (NDVI), and Yield (YLD) were transferred to improve performance under moisture deficit conditions. In BC_1_F_1_, BC_2_F_1,_ and BC_2_F_2_ generations, the foreground selection was carried out to identify the plants with positive QTLs conferring drought tolerance and linked to traits NDVI, CT, TKW, and yield. The positive homozygous lines for targeted QTLs were advanced from BC_2_F_2_ to BC_2_F_4_ *via* the pedigree-based phenotypic selection method. Background analysis was carried out in BC_2_F_5_ and obtained 78-91% recovery of the recurrent parent genome in the improved lines. Furthermore, the advanced lines were evaluated for 2 years under drought stress to assess improvement in MABB-derived lines. Increased GWPS, TKW, and NDVI and reduced CT was observed in improved lines. Seven improved lines were identified with significantly higher yields in comparison to HD3086 under stress conditions.

## Introduction

Wheat is the most important food grain grown worldwide as well as in India. On average, 35% of the world’s population depends on wheat as their staple food (IDRC, 2010). More than two-thirds of the world’s wheat is used for human consumption, while one-fifth is used for animal feed (Grote et al., 2021). It is estimated that a 60% increase can be expected in the consumption of wheat-based products by 2050 due to an expanding global population and food demand. In order to meet global demand, wheat yields need to be increased by 1.6% per year (Tilman et al., 2011). However, the average yield of wheat worldwide is much lower than its potential (Asseng et al., 2020; Rashid et al., 2022). Due to change in climatic conditions, wheat crop faces challenges like biotic and abiotic stress, which significantly reduce crop yield. New diseases and pests have been posing a significant threat to wheat production; coupled with increasing drought and heat stress due to changing environmental conditions (Gupta et al., 2010). Among abiotic stresses, recurrent drought has a major impact on agriculture through alteration in the phenology of crops and changes in diseases and insect dynamics, which ultimately reduces the potential yield (Daryanto et al., 2016; Nalley et al., 2018). Currently, 70% of the wheat-growing area worldwide experiences moisture deficiency stress (Portmann et al., 2010); nearly 50% of the wheat grown in developing nations is rain-fed, receiving an average of 600 mm of precipitation per year, with occasional lows of 350 mm. Additionally, only 1-2 irrigations are given to 66 - 80% of the wheat grown in irrigated conditions, which results in a reduction in yield (Joshi et al., 2007). It is anticipated that annual precipitation will fall by 4–27% in various parts of the world and the temperature will hike by 1.5°C as a result of global warming over the next 10 years (IPCC, 2021). These two abiotic stresses hamper production because they frequently coexist throughout the grain-filling stage in dry or semi-arid environments (Wardlaw and Willenbrink, 2000; Sallam et al., 2015). Wheat crop yield loss due to heat and drought can reach up to 86% and 69%, respectively (Prasad et al., 2011; Yang et al., 2021). Drought is an inadequacy of water, including precipitation and stored soil moisture, required for crop growth, both in terms of distribution and quantity, which results in a restricted expression of the genetic yield potential (Sinha et al., 1986).

The North Western Plains Zone and North Eastern Plains Zone of India together contribute 78 MT of wheat production in an area of 21 mha. In recent years, the ICAR-Indian Agriculture Research Institute (IARI) has contributed several high-yielding wheat varieties, including HD2967 and HD3086, which together account for 40% of the nation’s total wheat-grown area. In Indo-Gangetic plains, the high-yielding wheat variety HD3086 has been made available for commercial production under timely sown, irrigated conditions. It alone accounts for 11.6% of the wheat varieties indent and 34% of breeder seed indent in India (https://seednet.gov.in/). Due to its adaptability and higher yield, the breeder seed requirement for this variety is growing every year. However, its potential yield is reduced under moisture deficit stress conditions. According to previous reports, 68-70% of arable Indian land is under drought stress, especially in the wheat growing belt, including NWPZ and NEPZ, due to erratic rainfall and depleting stored water content (Mondal et al., 2017) leading to reduced wheat yield.

Drought tolerance is a complex trait; expression is controlled by polygenes and is influenced by various environmental factors (Gupta et al., 2012; Farooq et al., 2014). The complex inheritance mechanism of stress tolerance traits, limited genetic diversity of yield components under stress conditions, and the dearth of efficient selection techniques limit traditional breeding strategies for developing drought-tolerant varieties (Gupta et al., 2012; Gupta et al., 2017; Salarpour et al.),. An understanding of the genetic architecture of drought-related traits and information on relevant candidate genes/QTLs to develop drought-tolerant cultivars are necessary. In the past, many QTLs/meta-QTLs for yield and associated traits have been identified in wheat under drought stress which accounts 19-59% of the phenotypic variance (Quarrie et al., 2006; (Kirigwi et al., 2007; Shukla et al., 2015; Tahmasebi et al., 2016; Salarpour et al., 2021; Gupta et al., 2017). Apart from this, drought-related QTL mapping studies were also conducted under drought stress (Salarpour et al., 2021 and Salarpour et al., 2020) and heat stress conditions (Tahmasebi et al., 2016) in wheat. Early heading and anthesis, canopy temperature (CT), normalized difference vegetative index (NDVI), water-soluble carbohydrates (WSC), and chlorophyll content are key target agronomic and physiological traits for enhancing wheat crop ability to withstand drought (Bayoumi et al., 2008; Dolferus, 2014; Naeem et al., 2015; Afzal et al., 2017; Shamuyarira et al., 2019; Sobhaninan et al., 2019). Due to the complex inheritance pattern of drought tolerance, conventional breeding for these traits is difficult and time-consuming, hence schemes including quantitative trait loci (QTL) mapping and marker-assisted breeding should be used (Bahari et al., 2014). Additionally, it has been demonstrated that traditional breeding, in conjunction with marker-assisted selection (MAS), is effective for breeding complex traits, such as resistance to biotic and abiotic stresses, in a variety of crops, including wheat (Bustos et al., 2001; Gupta et al., 2010; Kumar et al., 2010; Kumar et al., 2011; Tyagi et al., 2014). The wheat variety DBW43 is known to perform better under moisture deficit conditions and also carries genes for leaf rust and yellow rust resistance. Hence, in the present study, DBW43 was used as a donor parent to transfer QTLs linked to component traits of drought tolerance in the background of HD3086 using marker-assisted backcross breeding (MABB).

## Materials and methods

### Plant materials and generation of improved lines

In the present MABB scheme, drought-tolerant germplasm line DBW43 was used as a donor parent, whereas HD3086 was used as the recurrent parent. Recurrent parent HD3086 is a popular high-yielding variety with a pedigree “DBW14/HD2733//HUW468”. It has semi-erect growth (99-101cm), has a 143-day period to maturity, and provides an average yield of 5.46 t/ha, but its potential yield is reduced under moisture deficit stress conditions. However, the donor parent DBW43 is the introduced line from CIMMYT, with pedigree “BABAX/*Lr42*//BABAX*2/3/VIVITSI”, and is identified at IIWBR, Karnal. The variety has an erect growth habit (110 cm) and is known to perform well under moisture deficit conditions; it also shows resistance to some races of yellow and leaf rust. Furthermore, DBW43 is a moisture deficit stress and heat-tolerant germplasm line; it is highly resistant to yellow and leaf rust and provides an average yield of 49 q/ha in RI conditions (Harikrishna, 2017; Singh et al., 2016).

**Figure 1:**
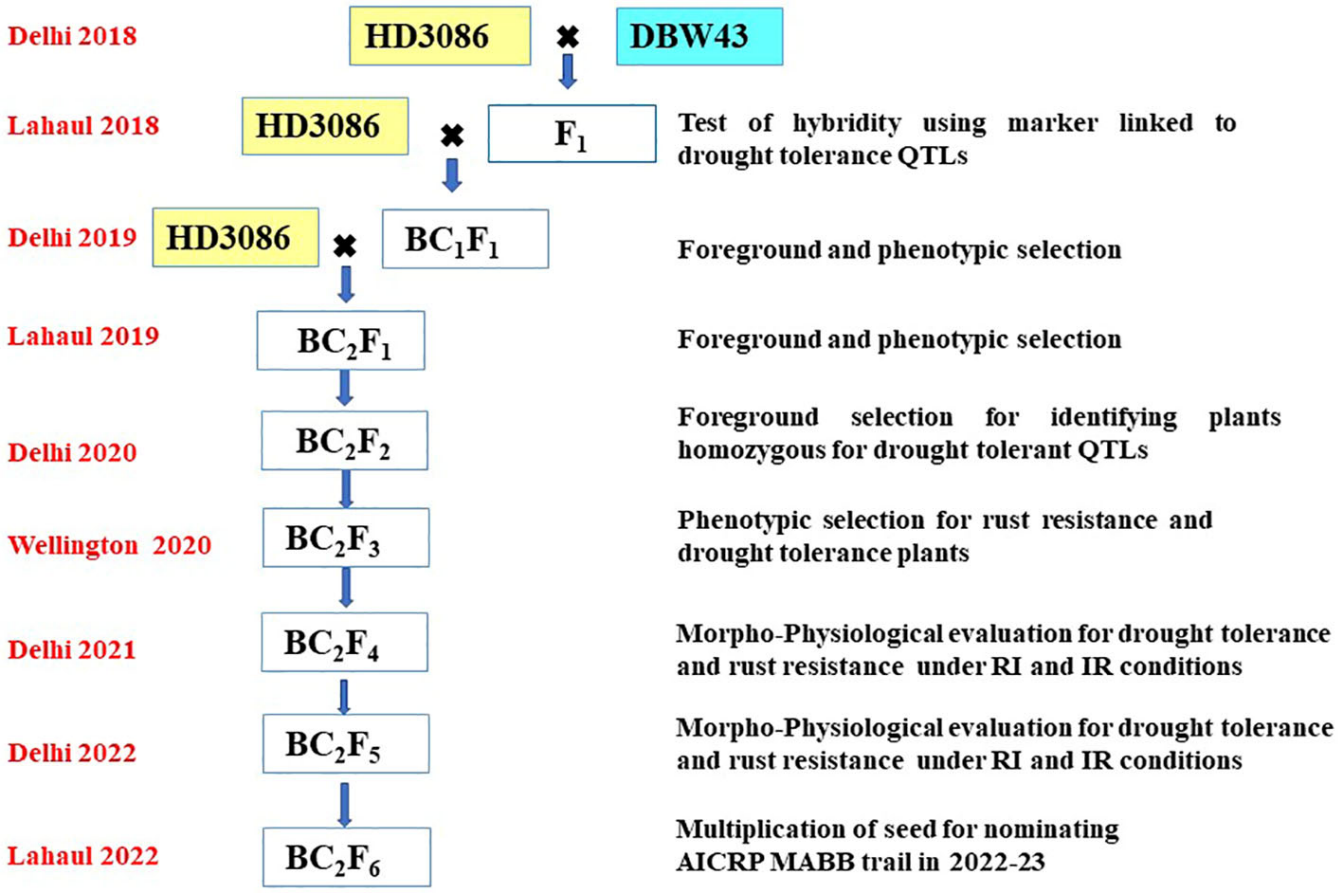
Schematic workflow of marker-assisted backcross breeding of HD3086*2/DBW43; the crossing pattern, season, and location of advancement and steps followed in each season are given in the figure.

The F_1_ plant’s hybridity was tested to check true F1s based on the presence of the hybrid band, using the linked SSR marker X*gwm484*. X*gwm484* was a polymorphic marker between parents and also a linked marker with the QTL related to drought-tolerant traits such as BIO, WSC, and Yield. True individual F_1_ plants were backcrossed twice with the recurrent parent HD3086 to generate BC_1_F_1_ and BC_2_F_1._ The following generations were handled by the procedure shown in **Figure 1**. The scheme comprises selection in each backcross generation, the foreground selection was performed with SSR markers linked to targeted drought-tolerant QTL regions (**Table 1**), followed by phenotypic selection (PS) for the plants similar to the HD3086 recurrent parent phenome (RPP). Foreground selection was undertaken in BC_1_F_1_, BC_2_F_1,_ and BC_2_F_2_ (**Table 2**). In each step, foreground screening for QTLs was carried out to select the plants before making backcross in F_1_ and BC_1_F_1_. The BC_2_F_1_ plants were also screened for drought-tolerant QTLs, and the selected plants were subjected to selfing to generate BC_2_F_2_ progenies. Furthermore, the selected homozygous BC_2_F_2_ progenies for targeted QTLs were advanced *via* pedigree-based phenotypic selection up to BC_2_F_4_ generation. The advancement of improved lines was done in New Delhi during the main season and in off-season nurseries such as Wellington and Lahul-Spiti. In each advanced generation, the phenotypic selection was done to achieve maximum recovery of the recurrent parent phenome. The selected BC_2_F_4_ and BC_2_F_5_ lines were tested for two years for tolerance to moisture stress in field conditions. The BC_2_F_5_ individuals that performed better than the recurrent parent for targeted traits were advanced to seed multiplication for nomination under the MABB trials of the All India Coordinated Research Projects (AICRP).

**Table 1:** Sequence of SSR primers and their amplification details for linked SSR markers used to transfer drought-tolerant QTLs in HD3086*2/DBW43 population.

SL.NO	Gene/Marker	Chromosome	Primer sequence (5’ – 3’)	Position (cM)	Annealing temperature (°C)	PVE (%)	Product size (bp)		Associated/Reported Traits	Reference
							HD3086	DBW43		
**1**	*Xgwm484*	2D	F - ACATCGCTCTTCACAAACCC R - AGTTCCGGTCATGGCTAGG	45	55	9.45 Kadam et al. (2012)	160	180	BIO, WSC, YLD	Yang et al. (2007)
**2**	*Xwmc617*	4A	F - CCACTAGGAAGAAGGGGAAACT R - ATCTGGATTACTGGCCAACTGT	13	61	14 (Harikrishna, 2017)	220-230	230-260	YLD	Pinto et al., 2010
**3**	*Xwmc640*	3A	F - AATTTATCTCGATCATGTGAGC R - TGAGTAGTTCCCTTAGGACCTT	43-53	61	14.01 Gu et al. (2015)	130 180	140	TKW, CT, DM (MQTL24)	Acuña‐Galindo et al. (2015)

**Table 2:** Number of plants selected in each generation in HD3086*2/DBW43 population.

Generation	No. of plants	Foreground Selection	
		No. of plants selected	
BC_1_F_1_	98	44	FS+PS
BC_2_F_1_	160	25	FS+PS
BC_2_F_2_	830	140	FS+PS
BC_2_F_3_	140	120	PS
BC_2_F_4_	120	42	BS+PS
BC_2_F_5_	10	7	PS
BC_2_F_6_	7	1	PS

FS, Foreground selection; PS, Phenotypic selection.

### Foreground selection

The techniques outlined by Hospital and Charcosset (1997) were used for foreground selection. The linked QTLs to drought-tolerant traits such as NDVI, CT, CC, DH, and Yld were used in MABB. SSR markers linked to these QTLs were validated using the RIL population generated by a cross between HI1500 x DBW43 (Harikrishna, 2017). The QTL Qndvi6.iari-4A with the phenotypic variance of 14% (R^2 = ^0.14) linked to SSR marker *Xwmc617*, a CT and TKW related meta QTL ‘MQTL24’ located on 3A linked to SSR marker *Xwmc640* with PV of 14.01%, and a biomass, WSC, and yield-related QTL located on 2D linked to SSR marker *Xgwm484* with a PV of 9.45% (Yang et al., 2007; Pinto et al., 2010; Kadam et al., 2012; Acuñ-Galindo et al., 2015; Gu et al., 2015; Harikrishna, 2017) were used in this study to transfer respective traits.

Genomic DNA was isolated from fresh leaf tissue using the CTAB procedure, DNA was quantified using a Nanodrop, and purity was tested using 0.8% agarose gel electrophoresis with λ DNA as a standard. PCR reaction was performed with a volume of 15 µl comprising 30-40 ng of template DNA, 5 pmol of each primer, 0.05 mM dNTPs, and 10X PCR buffer (10 mM Tris, pH 8.4, 50 mM KCl, 1.8 mM MgCl2, and 0.5 U of *Taq DNA polymerase*) (Bangalore Genei Pvt. Ltd., India). SSR markers from the *Xgwm* and *Xwmc* series were used to perform PCR amplification of the template DNA (**Table 1**). The amplified product was resolved in gel electrophoresis using 3% Agarose SFR gel and observed on Gel documentation systems (**Supplementary Figure 1**).

### Background analysis

To identify plants with the maximum recovery of the recurrent parent genome (RPG), selected BC_2_F_5_ lines along with parents were genotyped utilizing hybridization-based Wheat Breeder’s 35K Axiom Array SNP chips of the Affymetrix GeneTitan^R^ system. This array contained 35,143 SNPs that were evenly dispersed throughout the wheat genome. However, after filtering for monomorphic alleles, minor allele frequency (MAF), and missing data, a total of 3706 polymorphic SNPs were chosen between parents DBW43 and HD3086 and were used for background analysis. Using the GGT 2.0 (Graphical Geno Typing 2.0) software, the background recovery of the recurrent parent was graphically visualized (Van Berloo, 2008). The following formula was used to determine the recurrent parent’s contribution to the background of MABB-generated lines:

G = [(B + 1/2A) × 100]/N

were,

N = total number of parental polymorphic markers screened

B = number of markers showing homozygosity for recurrent parent allele

A = number of markers showing heterozygosity for parental alleles.

### Screening for leaf rust resistance at the seedling stage

In each backcross population, HD3086*2/DBW43 and parents were tested at the seedling stage for resistance to *P. triticina* races 77-5 and 77-9 using Single Race Testing (SRT) at IARI New Delhi and IARI Regional Station, Indore. Seedlings, at approximately 8–10 days old were inoculated with spores/suspension early in the evening. After inoculation, seedlings were raised under a humid glass chamber for 36 hours and maintained an ambient temperature of 23 ± 2°C and 85% relative humidity. Leaf rust resistance was scored for each race using the 0–4 modified Stakman Scale (Roelfs, 1992; **Table 3**). The disease reaction was recorded 12–14 days after the inoculation.

**Table 3:** Leaf rust scoring method according to modified Stakman scale (Roelfs, 1992).

Infection types	Leaf rust response/reaction	Disease response
0	No flecks or uredinia	Immune
0;	faint Hypersensitive flecks	Highly resistant
;	Hypersensitive flecks	Highly resistant
1	Small uredinia with necrosis	Resistant
2	Small to medium uredinia with necrosis	Moderately susceptible
3	Moderate to large size uredinia with/without chlorosis,	Susceptible
4	Very large uredinia without chlorosis	Highly susceptible
X	Mesothetic, a mixture of resistant pustule types	Mesothetic
“+”	Indicates slightly larger uredinia	–
“-”	Indicates slightly smaller uredinia	–

If more than one kind of reaction is observed in a plant, they are written with consecutive scores, For example, if a plant has both 1 and 2 reactions, it is written as “1 2”. Similarly, if a line has; and 1 reactions, it is written as “; 1”

### Screening of genotypes at the adult stage for leaf and yellow rust resistance

Field screening for leaf and stripe rust was done in each backcross generation at IARI, New Delhi during the main season, and in off seasons at Wellington and Shimla. “Agra local”, a rust-susceptible landrace, served as the infector and was planted around the experimental plots after every 20 rows of backcross population were planted for screening. The lines under testing were sprayed with a mixture of urediospores from the prevalent stripe and leaf rust pathotypes to create artificial rust epiphytotic. To guarantee uniform disease propagation, rust-infected pots were positioned in fields between the experimental materials. The infected leaves of the susceptible host were suspended in water containing urediniospores (5.6 g/ha) and 0.75 l/ml Tween20 (surfactant) following the method described by Imtiaz et al. (2003). According to the modified Cobb scale provided by Peterson et al. (1948), the disease severity (DS) and infection response (IR) were recorded at the reproductive stage. The DS was expressed in percentage (0-100%) and the IR was recorded as S (susceptible), MS (moderately susceptible), MR (moderately resistant), and TR (trace).

### Evaluation for drought tolerance traits

The selected backcross populations were evaluated for agronomic and physiological parameters for two years at the IARI experimental farm in New Delhi (280 40’N, 770 13’E, MSL228m). In the year 2020-2021, parents (viz., HD3086, DBW43), three check varieties (viz., HI1500, GW322, and BABAX), and the selected positive BC_2_F_4_ lines were evaluated in an augmented design under irrigated conditions (IR) and restricted irrigation (RI) conditions. Irrigated trials received a total of six irrigations, whereas restricted irrigated trials received only one irrigation (21 days after sowing, in addition to pre-sowing irrigation) to induce terminal drought stress. The experiment was conducted in the same field with a divider to restrict irrigation water flow to the RI treatment. Weather data during the growing season of wheat (November to March) during 2020-2021 and 2021-2022 are included in **Supplementary Table 1**. A plot size of 0.63 m^2^ was maintained, in which each plot consisted of three rows with a spacing of 23 cm between rows. The BC_2_F_5_ lines, which had performed better in the previous generation, were evaluated in RCBD (randomized complete block design) using large plots of size 7.2 m^2^ (6 m x 1.2 m) under IR, RI, and late sown (LS) conditions in the year 2021-22. The wheat crop was raised using standard agronomic management techniques.

The “Wheat Physiological Breeding II: A Field Guide to Wheat Phenotyping” (Pask et al., 2012) manual was used as the guide for the standard method of data collection in improved lines (**Supplementary Table 2**). Agronomic traits like days to heading (DH), days to maturity (DM), plant height (PH), plot yield (PY), thousand kernel weight (TKW), and grain weight per spike (GWPS) were recorded from each entry. Physiological parameters including NDVI, CT, and soil plant analysis development (SPAD) chlorophyll content were measured at three distinct stages, namely, the vegetative stage (late boot stage, Z49), grain filling stage (early milk stage, Z73), and grain maturity stage (late milk stage, Z85) (Zadoks et al., 1974).

### Statistical analysis

Descriptive statistics and analysis of variance were calculated for BC_2_F_4_ individuals using an R package ‘augmented RCBD’ (Aravind et al., 2021). Comparisons of individuals were conducted based on adjusted mean calculated with the formula (Federer, 1961)

Vi=ui−bj Where

Vi is the Adjusted mean of *i^th^* variety

*ui* is the Unadjusted mean of *i^th^* variety

*bj* is *j^th^* block effect

Whereas the BC_2_F_5_ lines were planted in RCBD with three replications, and the analysis of variance, Least Significant Difference (LSD), and Coefficient of Variation (CV) were calculated from MS Excel following standard procedure. The lines that significantly performed better under moisture deficit stress were identified.

## Results

### Development of backcross population following foreground selection and background analysis

#### Foreground selection for drought-tolerant QTLs

To transfer the drought tolerance QTLs, the recurrent parent HD3086 was crossed with the donor parent DBW43, and BC_1_F_1_ progenies were produced. The true hybrid plants from the cross were backcrossed with recurrent parent HD3086 to enhance the recurrent genome portion in the progenies. A total of 98 BC_1_F_1_ plants were screened for the presence of different foreground markers linked with QTLs of interest. Among them, 44 plants were found to contain positive alleles for required QTLs and were selected for the second round of backcrossing. To generate BC_2_F_1_, the selected plants were again backcrossed with the recurrent parent HD3086. Among 160 BC_2_F_1_ plants, 25 plants were positive for targeted drought tolerant QTLs and were selfed to develop BC_2_F_2_ progenies. All the 830 BC_2_F_2_ plants obtained were grown and homozygosity for the markers linked with donor QTLs was identified from foreground selection. 140 BC_2_F_2_ homozygous plants were selected *via* foreground selection with a different combination of QTLs related to drought tolerance and were advanced up to BC_2_F_4_ through pedigree-based phenotypic selection.

### Background recovery of the recurrent parent genome

**Figure 2:**
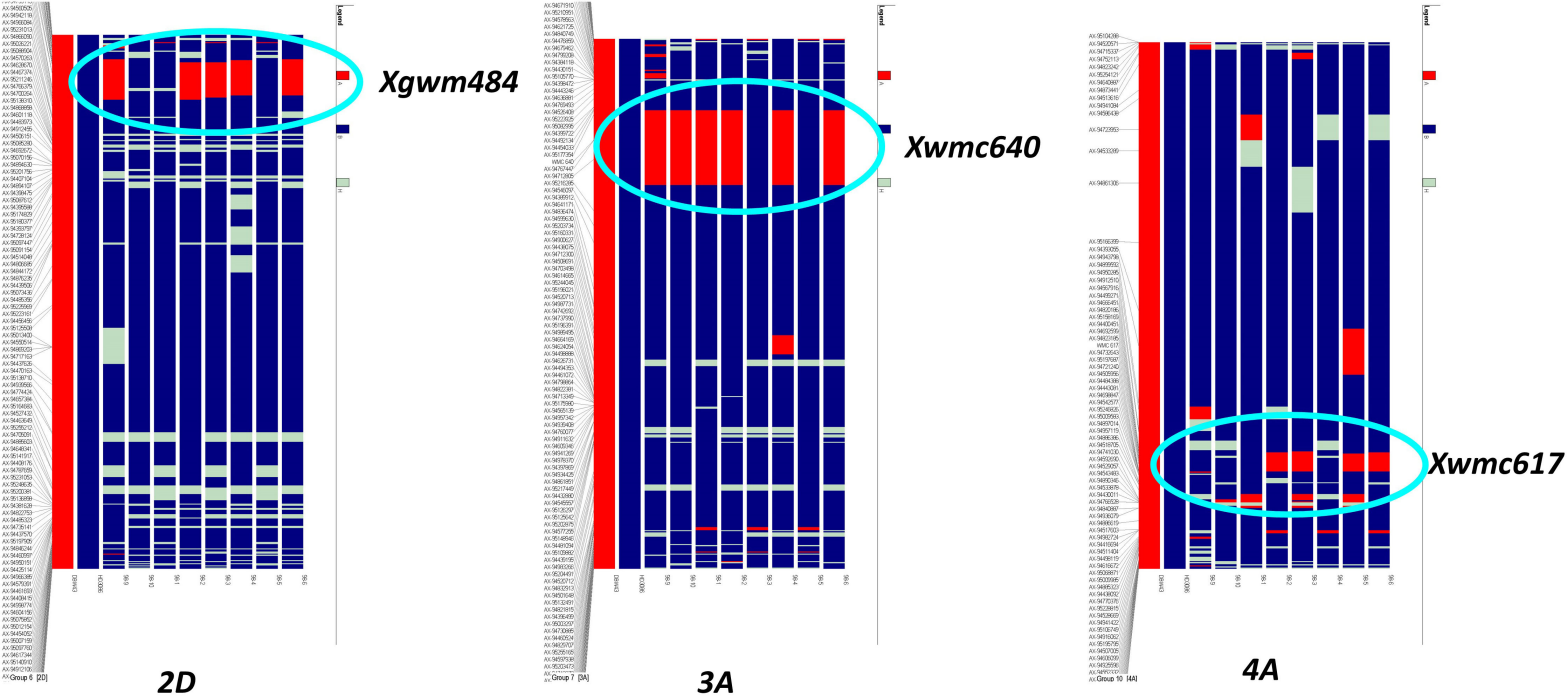
Recurrent parent recovery of chromosomes 2D, 3A, and 4A; first two chromosomes indicate donor (red) and recipient (blue) chromosomes followed by progeny. The red portion indicates donor genome segment; the blue portion indicates recipient genome segments. Chromosomal location and donor segment linked to SSR markers *Xgwm484*, *Xwmc640*, and *Xwmc617* are shown with circles.

Improved BC_2_F_5_ homozygous lines were subjected to background analysis for recovery of the recurrent parent genome, and eight lines were identified with maximum recovery of the recurrent parent genome along with drought-tolerant QTLs from the donor (**Figure 2**). The recurrent genome recovery of improved lines ranged from 78%-91%, with an average recovery of 85.5%. Improved lines had maximum visual similarity at the phenotypic level with recurrent parent HD3086. Among these, two lines, viz., HD3086-7-1-210-26 and HD3086-3-15-174-22, had a maximum recurrent parent genome recovery of 92% and 90%, respectively. The targeted QTLs from the donor parent, which are spread over 3A, 4A, and 2D chromosomes, were transferred to the progenies developed in this study (**Figure 2**).

### Phenotypic screening for rust resistance

Single race testing (STR) for leaf rust resistance at the seedling stage confirmed a similar susceptibility reaction of recurrent parent HD3086 to that of check Agra local (3 3^+^) for both pathotypes, 77-5 and 77-9. However, donor parent DBW43 had a Highly Resistant (HR) response (0); for pathotypes 77-5 and 77-9 (**Table 4**). Furthermore, 15 BC_2_F_5_ drought-tolerant lines were subjected to SRT for *P. triticina* pathotypes 77-5 and 77-9, of which 11 lines showed resistance reactions (such as 3HR, 3R, and 5MR), and the rest of the 4 lines displayed susceptible reactions at the seedling stage. Among these improved lines, HD3086-5-1-189-24, HD3086-6-6-209-25, and HD3086-13-10-279-34 showed a highly resistance reaction, which is similar to the resistance of parent DBW43.

**Table 4:** Screening of marker-assisted derived wheat genotypes for leaf rust races at IARI New Delhi and IARI regional station, Indore (BC_2_F_5_).

Sl.no	Progenies	Rust race (77-5)		Rust race (77-9)		Reaction of genotypes
		IARI RS, Indore	IARI, New Delhi	IARI RS, Indore	IARI, New Delhi	
1	HD3086 (Recipient parent)	33^+^	3	2 3	3	Susceptible (S)
2	DBW43 (Donor parent)	0;	0	0;	0;	Highly Resistant (HR)
3	HD3086-1-3-126-21	3	0	3	0	S
4	HD3086-3-15-174-22	; 1	1	; 2	;	R
5	HD3086-4-4-184-23	;	; 1	; 2	2	MR
6	HD3086-5-1-189-24	;	;	;	1	HR
7	HD3086-6-6-209-25	; 1	1	; 1	;1	HR
8	HD3086-7-1-210-26	0	1 2	; 1	2	MR
9	HD3086-7-3-212-27	; 1	1	; 1^-^	1 2	R
10	HD3086-7-6-215-28	; 1	1 2	; 2	1 2	MR
11	HD3086-7-7-216-29	; 1	;	; 2	; 1	R
12	HD3086-7-11-220-30	0;	2	3	1, 2	MS
13	HD3086-10-1-244-31	0	0, 1	3^+^	0,;	MS
14	HD3086-11-6-257-32	; 2	1;	; 2	1 2	MR
15	HD3086-13-6-275-33	0;	1	2 3	; 1	MR
16	HD3086-13-10-279-34	0;	0;	;	1	HR
17	HD3086-15-10-293-35	3^+^	1	; 2	0	S
18	Agra Local	3+	3+	3+	3+	HS

HR, Highly resistant; R, Resistant; MR, Moderately resistant; MS, Moderately Susceptible; S, Susceptible; HS, Highly susceptible.

Backcross-derived lines (BC_1_F_1_, BC_2_F_1_, BC_2_F_2_) with positive drought-tolerant QTLs and resistance to leaf and stripe rust were advanced to the next generation. In BC_2_F_3,_ the rust severity of progenies was assessed along with the parents. The recurrent parent HD3086 showed a severity of 60S DS for leaf rust, whereas improved lines showed 0 to 20S DS. Improved lines for drought tolerance and complete resistance to leaf and stripe rust were selected in advanced generations.

### Morpho-physiological performance of the selected lines for drought tolerance

The improved lines from the cross HD3086*2/DBW43 carrying drought tolerance QTLs were evaluated for 2 years under IR and RI conditions. In the year 2020-2021, a total of 120 BC_2_F_4_ lines were evaluated in an augmented design, among which, 42 lines were found significantly superior to the recurrent parent and checks for introgressed traits. Furthermore, these 42 superior BC_2_F_4_ lines were evaluated for component traits of drought tolerance, and seven improved lines superior to recurrent parent HD3086 and the maximum recurrent parent phenome were identified. In the current study, leaf chlorophyll index (LCI) in BC_2_F_5_ ranged from 44.8 to 52.4, and approximately seven progenies outperformed the recurrent parent HD3086 in terms of LCI. The NDVI value of these selected lines ranged from 0.34 to 0.55, with a mean value of 0.45, which is more than the mean value (0.32) of recurrent parent HD3086 during the grain maturity stage. Eight progenies show lower canopy temperature than the recurrent parent; the mean CT value was 32.8 °C and ranged from 32.1°C to 34.4°C. Similarly, improved lines showed superior agronomic performance for the traits like TKW, GWPS, and yield under RI conditions (**Table 5**).

**Table 5:** Morpho-physiological characteristics of HD3086*2/HI1500 selected BC_2_F_5_ lines for component traits of drought tolerance under moisture deficit stress condition.

S.no	Selected progeny	QTLs linked trait	Chlorophyll content		Canopy temperature (°C)			NDVI			GWPS (g)	TKW (g)	YLD (q/ha)	Total RPG %
			VS	GFS	VS	GFS	GMS	VS	GFS	GMS				
1	HD3086-1-3-126-21	CT	49	50	25.6	30.3	32.9	0.78	0.65	0.34	1.24	33.5	44.18	78.31
2	HD3086-3-15-174-22	NDVI, TKW, YLD	50.5	51	24.9	28.8	32.5	0.79	0.73	0.48	1.63	38.1	49.58	89.81
3	HD3086-4-4-184-23	NDVI, BIO, YLD	44.8	48.9	25.3	30.6	34.4	0.77	0.7	0.45	1.61	34.8	45.57	86.47
4	HD3086-5-1-189-24	BIO, GWPS	47.9	51.1	24.7	30	33.5	0.75	0.67	0.51	1.53	33.2	49.17	88.46
5	HD3086-6-6-209-25	TKW, YLD	48.8	51.2	25.3	29.5	32.3	0.81	0.72	0.55	1.69	27.6	36.98	80.28
6	HD3086-7-1-210-26	NDVI, TKW, YLD	48.5	52.4	25.8	29.3	32.1	0.77	0.62	0.53	2.09	42.3	50.28	91.31
7	HD3086-7-11-220-30	BIO	46.7	49.5	27.6	32.5	32.3	0.75	0.64	0.4	1.47	36	40.30	83.47
8	HD3086-10-1-244-31	GWPS, CT	48.1	49.8	27.3	32.1	32.1	0.75	0.56	0.44	1.38	36.5	43.35	87.94
9	HD3086-11-6-257-32	NDVI, TKW	48.6	49.6	27.1	30.3	33.5	0.78	0.69	0.45	1.83	32.7	46.54	83.75
10	HD3086-13-10-279-34	CT, TKW	46.3	49.6	27.7	30.5	32.9	0.76	0.66	0.4	1.17	34.4	41.00	85.43
	HD3086(RI)		48.5	49.8	29.1	31.3	33.2	0.78	0.61	0.32	1.01	30.4	42.66	
	HD3086(IR)		49.4	52.6	22	31.3	32	0.79	0.72	0.54	1.65	44.6	62.60	
	DBW43(RI)		50.1	52.3	26	30.5	31.9	0.75	0.66	0.5	1.58	36.5	49.86	
	DBW43(IR)		51.2	52.5	22.7	28.6	31.6	0.76	0.7	0.58	2.01	39.7	53.88	
	Mean		47.98	50.43	26.37	30.48	32.80	0.77	0.66	0.45	1.52	34.67	44.96	
	LSD at 5%		2.93	2.84	1.39	1.33	2.18	0.03	0.03	0.02	0.09	1.95	2.32	

LSD, Least Significant Difference. NDVI, Normalized Difference Vegetation Index; GL, Grain Length; GWPS, Grain Weight Per Spike; TKW, Thousand Kernel Weight; YLD, Yield; RPG, Recurrent Parent Genome; RI, Rainfed; IR, Irrigated; VS, Vegetative stage; GFS, Grain Filling Stage; GMS, Grain Maturity Stage.

**Figure 3:**
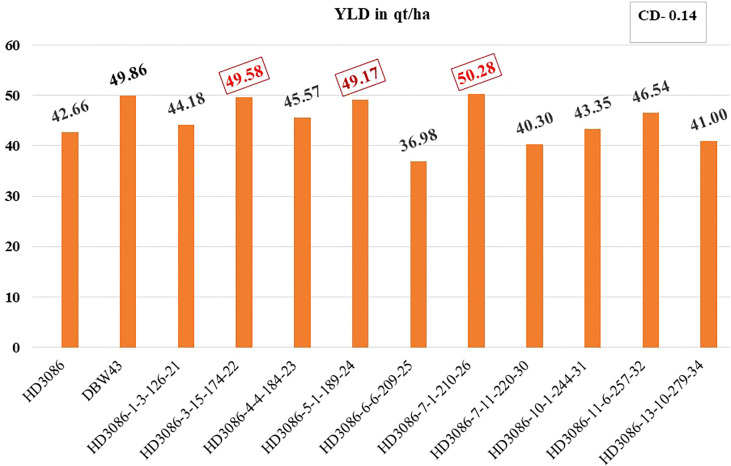
Improvement in grain yield of selected lines of HD3086*2/DBW43 derived BC2F5 population under moisture stress. Grain yield performance of parental lines (HD3086 and DBW43) along with the improved lines are given in quintals per hectare.

The distinctiveness, uniformity, and stability (DUS) of selected BC_2_F_5_ lines were assessed. Finally, 3 out of 10 progenies (viz., HD3086-6-6-209-25, HD3086-7-11-220-30, and HD3086-13-10-279-34) showed lower performance than the recurrent parent, and the rest of the seven progeny lines (*viz*., HD3086-1-3-126-21, HD3086-3-15-174-22, HD3086-4-4-184-23, HD3086-5-1-189-24, HD3086-7-1-210-26, HD3086-10-1-244-31, and HD3086-11-6-257-32) performed better in both the years than recurrent parent HD 3086. Furthermore, they were found to have a maximum recovery of both RPG and RPP and were further nominated for All India Coordinated Wheat Improvement Project (AICWIP) trials (**Figure 3**). Recently, in 2022-23, the HD3086-7-1-210-26 derived line was proposed for national trials for testing and further release for commercial cultivation under the designation HD3470.

## Discussion

Abiotic stress, particularly heat and drought, significantly declines wheat productivity. The ability to absorb nutrients is significantly affected by drought stress, which results in stunted growth and low yields in arid zones (Yasmin et al., 2019). Drought stress causes DNA, lipid, and membrane damage as well as the generation of reactive oxygen species in mitochondria, peroxisomes, and chloroplasts, which ultimately leads to the destruction of plant metabolism (Rashid et al., 2022). The majority of traits related to better performance under drought stress, such as RWC, days to heading, awn length, and spikelets per spike, had a positive relationship with yield (Sobhaninan et al., 2019) and are quantitatively inherited and governed by many genes/QTLs. Thousands of QTLs for agronomic and yield-contributing traits have been identified under different stress conditions in several previous studies (reviewed in Pinto et al., 2010; Gupta et al., 2012; Gupta et al., 2017; Shashikumara et al., 2020; Devate et al., 2022). However, their particle utilization in plant breeding needs to be emphasized with marker-assisted breeding programs. MABB is one of the practical and affordable methods of marker-assisted selection (MAS) that involves the transfer of desired traits from a donor parent to an elite recurrent parent, with minimum changes in the recurrent parent’s genetic background (Collard and Mackill, 2008; Bapela et al., 2022). MAS has been utilized frequently in bread wheat for the introgression of traits, including disease resistance and quality enhancement (Kumar et al., 2010; Malik et al., 2015; Shah et al., 2017; Sharma et al., 2021). However, a small number of studies have previously transferred QTLs for promising wheat varieties to increase drought tolerance and yield under moisture deficiency conditions (Merchuk-Ovnat et al., 2016; Rai et al., 2018; Gautam et al., 2020; Todkar et al., 2020; Sunilkumar et al., 2022). Wheat variety HD3086 is the choice of millions of farmers in Indo-Gangetic plains; its potential yield is reduced under restricted irrigation (RI) conditions. To achieve this, the current study aimed to transfer QTLs linked to component traits of drought tolerance in the genetic background of HD3086 through MABB.

Foreground selection was carried out in BC_1_F_1_, BC_2_F_1,_ and BC_2_F_2_ using the markers linked to QTLs for component traits of drought tolerance, such as NDVI, CT, TKW, and yield. NDVI sensor enables quick ground-level measurements of crops with the resolution required to characterize the canopy for its biomass, nutrient content, leaf area, and green area indices (Prasad et al., 2011). The SPAD meter measures leaf chlorophyll index *via* light transmittance that is differentially observed by chlorophyll and estimates leaf chlorophyll content and nitrogen content (Weier and Herring, 2000; Araus et al., 2008). DH, CT at the grain filling stage, and TKW are the most commonly used traits as indirect selection to improve yield under drought and heat stress (Tahmasebi et al., 2014). Similarly, Kernel number, grain yield, and chlorophyll content have been found to co-localize with regions controlling other drought adaptation traits, such as canopy temperature, and lead to deeper root system development and help in greater water absorption under stress (Pinto et al., 2010; Pinto and Reynolds, 2015; Diab et al., 2008; Olivares-Villegas et al., 2008). Therefore, in our study, three QTLs (BIO, WSC, and YLD: *Xgwm484*; CT and YLD: *Xwmc617*; TKW, CT, DM (MQTL24): *Xwmc640*) associated with component traits of drought tolerance were successfully transferred in the genetic background of HD3086 using MABB and phenotypic selection. The presence of introgressed QTLs located on wheat chromosomes 2D, 3A, and 4A explained a phenotypic variance from 9.45% to 14.01% previously validated in our lab using the RIL population HI1500 x DBW43, Harikrishna (2017) and other independent studies (Kadam et al., 2012; Acuña‐Galindo et al., 2015; Gu et al., 2015). In the present study, a positive correlation between NDVI, LCI, and TKW with yield and a negative correlation between CT and DH with yield were observed. Similar correlation results were also reported by Lopes and Reynolds (2012), Harikrishna et al. (2016), and Ramya et al. (2016), indicating that productivity under moisture deficit stress was improved by the increased performance of component traits of drought tolerance, such as NDVI, chlorophyll content, and canopy temperature depression. In the current study, seven promising lines that perform better than their recurrent parent under RI conditions were developed. The two lines that possess QTL combinations for Biomass, TKW, and yield showed a 15-17% improvement in yield over recurrent parent HD3086. In a related earlier study by Rai et al. (2018), five prospective varieties were developed in the background of HD2733 by transferring NDVI, CT, and chlorophyll content linked QTLs by MABB and were found to perform well under rainfed conditions. Similar to this, wheat variety GW322 was improved for traits such as NDVI, stay green, chlorophyll content, and yield, and 18 superior BC_2_F_3_ drought-tolerant progenies were identified in advanced generations of MABB-derived lines (Todkar et al., 2020). In this study, along with drought tolerance, lines were also screened for rust resistance in each generation, and the final improved lines showing resistance to both leaf and stripe rust were selected. The donor germplasm line DBW43 has the *Lr42* gene in their pedigree, and recurrent parent HD3086 showed resistance to 78S84 and 46S119 pathotypes of stripe rust (Singh et al., 2014); this might be the reason the drought-tolerant improved lines in cross HD3086*2/DBW43 also showed resistance to both leaf and stripe rust.

The potential applications of MAS in wheat have been further expanded by the advent of high-throughput sequencing, precision phenotyping, crop molecular physiology, and computational tools. Wheat has the biggest genome size (~16GB; Somers et al., 2004), and it is difficult to cover entire genomic areas with uniformly spaced polymorphic SSR markers. Background selection was performed using 35k SNPs in BC_2_F_5_ along with parents and identified plants with a maximum RPG ranging from 78%-91%. SNPs are almost 300 times more cost-effective than SSR markers for background analysis; therefore, SNPs could be a better choice for background selection in MABB (Khanna et al., 2015). Through the combination of phenotypic selection and marker-assisted background selection, the genome of the recurrent parent HD3086 recovered more quickly and to a greater extent compared to the estimated average recovery percentage. It has been observed in previous research that two backcrosses with selection for RPP were sufficient for better genome recovery in important cereal crops like wheat (Xu et al., 2017; Sharma et al., 2021; Mallick et al., 2022; Sunilkumar et al., 2022), rice (Ellur et al., 2016; Grover et al., 2020), and maize (Hossain et al., 2018; Zunjare et al., 2018). In our study, two drought-tolerant improved lines, HD3086-7-1-210-26 and HD3086-3-15-174-22, had maximum recovery rates of 92% and 90%, respectively. Similar genome recovery rates, i.e., 89.2% to 95.4%, were observed by Rai et al. (2018) in MABB for drought tolerance QTLs in the background of HD2733. In a study containing gene pyramiding of leaf rust resistance genes into an elite cultivar, HD2687, 94.55% genome recovery rates in BC_2_ generations were observed by Bhawar et al. (2011). Similarly, 98.25% RPG recovery was observed in the transfer of gene *LrTrk* from *Triticum turgidum* cv. *Trinakria* to hexaploidy wheat variety HD2932 by Mallick et al. (2022) through MABB.

## Conclusion

The current work demonstrates the back-crossing breeding technique that combines phenotypic selection and marker-assisted selection to transfer QTLs related to drought tolerance in the background of the well-known wheat variety HD3086. We have successfully transferred three QTLs governing component traits under drought, viz, NDVI, CT, BIO, WSC, YLD, TKW, and DM, to develop improved lines that are performing well under drought stress using the MABB scheme. The transfer of QTLs led to the development of superior varieties in the genetic background of the existing variety HD3086. We have identified seven superior lines over the parent HD3086 under drought stress. The improved lines with more than 90% genomic similarity with the recurrent parent can be released as a superior variety over the existing variety HD3086 under restricted irrigation conditions. Millions of farmers in major wheat-growing regions choose wheat variety HD3086 because of its excellent yielding ability and quality. Furthermore, expanding the area of cultivation in NWPZ and NEPZ with limited irrigations is made possible by improving this variety for moisture deficit stress tolerance.

## Data availability statement

Data is uploaded to Dryad data base, available at following link. https://datadryad.org/stash/share/Bf6VjldDzhcIS0YNqyTRd_qVOllEckhCx1_-TkJK8YE.

## Author contributions

Conduct of experiment and writing of manuscript draft: VPS and HK; Supervision: PS, GS, and NJ; Phenotypic data collection: VPS, SS, DC, ND, and JS; Evaluation of rust: VPS, HK, PL, DP, and SM; Genotypic data: VPS, PS, HK, SS, DC, NS, and KM; Statistical analysis: VPS, ND, and HK. All authors contributed to the article and approved the submitted version.
