# Supplementary material for: Marker-assisted selection for transfer of QTLs to a promising line for drought tolerance in wheat (Triticum aestivum L.)
Source: Front Plant Sci. 2023 Jul 21;14:1147200. doi: 10.3389/fpls.2023.1147200 (PMC10401266; doi:10.3389/fpls.2023.1147200)
Supplement: Supplementary file 1 [file DataSheet_1.zip › Suppli fig 1.pptx]

## Slide 1
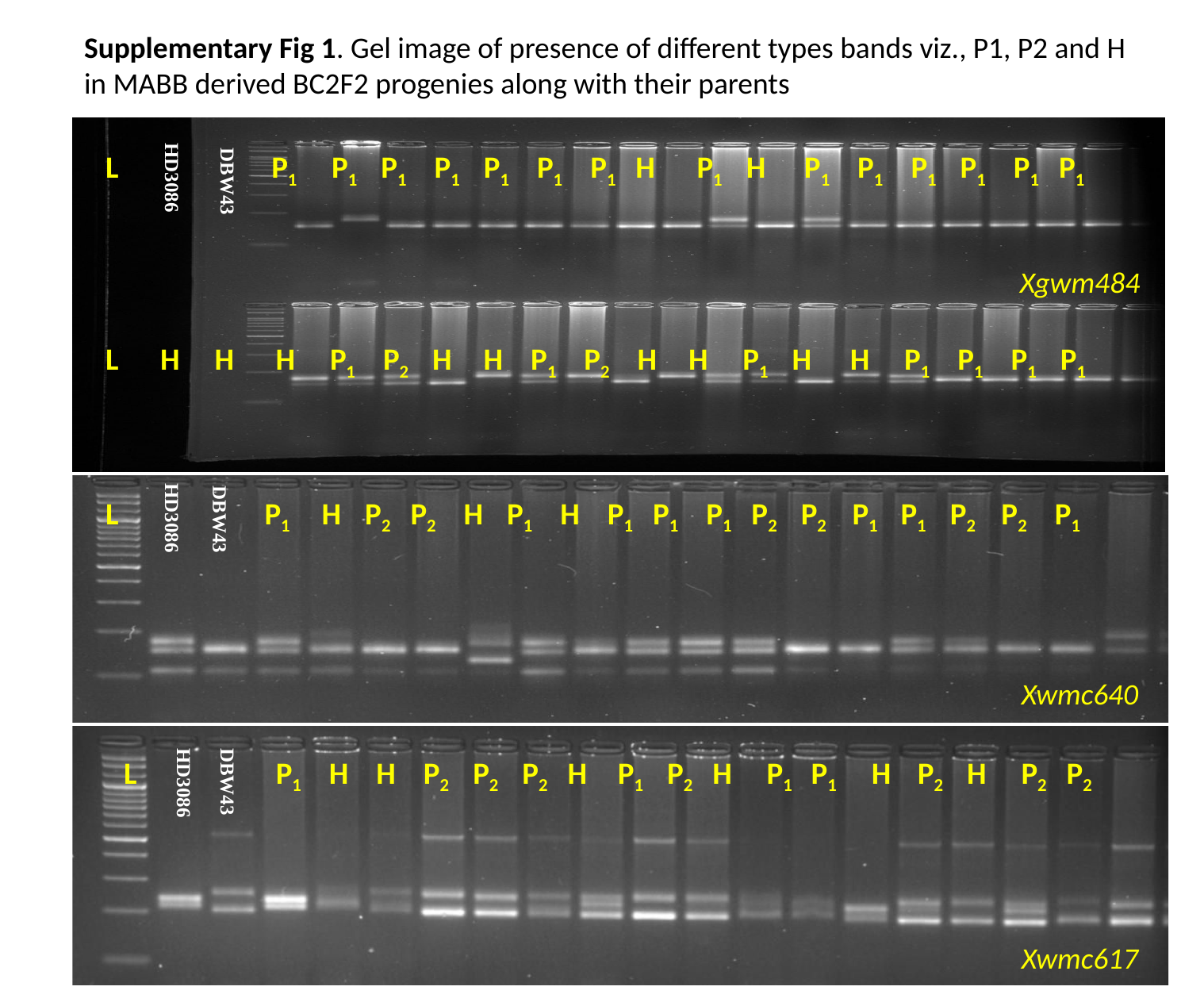

Supplementary Fig 1. Gel image of presence of different types bands viz., P1, P2 and H in MABB derived BC2F2 progenies along with their parents
 L P1 P1 P1 P1 P1 P1 P1 H P1 H P1 P1 P1 P1 P1 P1
HD3086
DBW43
Xgwm484
 L H H H P1 P2 H H P1 P2 H H P1 H H P1 P1 P1 P1
 L P1 H P2 P2 H P1 H P1 P1 P1 P2 P2 P1 P1 P2 P2 P1
HD3086
DBW43
Xwmc640
 L P1 H H P2 P2 P2 H P1 P2 H P1 P1 H P2 H P2 P2
DBW43
HD3086
Xwmc617
