# Supplementary material for: Marker-assisted selection for transfer of QTLs to a promising line for drought tolerance in wheat (Triticum aestivum L.)
Source: Front Plant Sci. 2023 Jul 21;14:1147200. doi: 10.3389/fpls.2023.1147200 (PMC10401266; doi:10.3389/fpls.2023.1147200)
Supplement: Supplementary file 1 [file DataSheet_1.zip › Suppli Tab 2.docx]

**Phenotyping**

The experimental materials were evaluated for important physiological and agronomical characters under IR, RI and LS conditions responsible for stress tolerance. The book "Physiological Breeding II: A field guide to wheat phenotyping" released by CIMMYT was used as reference to measure the targeted traits (Pask et al., 2012).

**1 Days to heading (Days)**

Days to heading is defined as number of days from the sowing date to the date on 50% heading. When 50% of the tillers began extrusion awns out of the flag leaves, the number of days needed to head the entire plant population was noted.

**2 Days to maturity:**

The physiological maturity was considered when plants turned pale yellow due to senescence. Number of days from sowing to senescence was recorded in each plot for this purpose.

**3 Plant height (cm)**

Five randomly selected plants at maturity were measured from the ground to the spike's tip using a wooden scale of 160 cm, excluding awns.

**4 Spike length (cm)**

Three randomly chosen plants' main tiller spikes were selected, and their lengths from base to tip, without considering the awns, were measured in cm at maturity.

**5 Thousand Kernel weight (g)**

A total of one thousand kernels were manually counted and their grains were weighed using an electronic digital balance. Unit expressed in grams.

**6 Grain weight per spikes (g)**

Randomly 20 spikes collected from different plants of each genotypes were threshed together and grains were weighed from an eclectic balance in grams. Unit expressed in grams per spike.

**7 Biomass (g)**

Plants from each plots were harvested by cutting at the ground level after drying and bulk weight was measured in grams for biomass. Unit expressed in grams.

**8 Grain yield (g)**

Each plot was harvested and threshed separately, and the grains were weighed on an electric balance. The grain yield is expressed in grams per plot.

**9 Canopy temperature (CT) (^0^C)**

Canopy temperature is a measure of cooling effect by evapotranspiration, and an indirect measure of water absorption from deeper soil layers. The temperature of the canopy of each plot was measured with a portable infrared thermometer (Kane May Model Infratrace 8000, USA) in ^0^C. The measurements were taken between 30^0^- 60^0^ from the horizontal, nearly 0.5 m from the plots edge, and approximately 60 cm above the canopy. Measurements were performed between 11:00 AM and 1:00 PM on clear, sunny days with a calm wind speed on the plant row side that receives the most sun exposure.

**10 Normalized difference vegetation index (NDVI)**

It is widely used to measure ground cover, vegetative greenness and canopy photosynthetic rate in different growth stages of the plant. NDVI was measured using Green Seeker, a field portable device, works on the principle of absorbance of red light (Red) by healthy green canopy and reflectance of near infrared (NIR) light. NDVI readings often decrease as a plant reaches maturity and subsequent senescence. A total of 4-5 readings were recorded at different growth stages and average of the three readings at a given stage was used as the basis for analysis.

NDVI = (NIR-Red) / (NIR+Red)

**11 Leaf Chlorophyll Index (LCI)**

The Minolta SPAD-502 chlorophyll meter, a hand-held battery-operated portable device, was used to measure chlorophyll content, an important indicator of photosynthetic rate. It works by absorbing red light at 650 nm and transmittance of infrared light at 940 nm. After the instrument had been calibrated, measurements were taken on completely expanded, dry, and unbroken flag leaves of the main tillers of the selected plants, avoiding thick areas like midribs and veins while positioning the instrument adaxial surface upwards. Five plants were chosen at random from each plot, and SPAD readings were taken from flag leaf of the plant at vegetative and grain filling stage.
